# Encephalitis: Predictive Role of Clinical and Diagnostic Data on Outcome—A Monocentric Study

**DOI:** 10.3390/life15081313

**Published:** 2025-08-19

**Authors:** Deborah K. Erhart, Luisa T. Balz, Hayrettin Tumani

**Affiliations:** Department of Neurology, University Hospital of Ulm, Oberer Eselsberg 45, 89081 Ulm, Germany; deborah.erhart@uni-ulm.de (D.K.E.); luisa.balz@uni-ulm.de (L.T.B.)

**Keywords:** encephalitis, infectious CNS disease, outcome, MRI, seizures, CSF findings, CXCL13

## Abstract

Encephalitis is a potentially life-threatening condition with long-term neurological sequelae. However, data on early clinical, demographic, and diagnostic predictors of functional outcomes remain limited. We performed a retrospective monocentric study including 98 patients diagnosed with infectious encephalitis of various etiologies treated in the University Hospital Ulm between January 2014 and December 2024. Ordinal logistic regression models were applied to evaluate associations between admission characteristics and functional outcome at discharge, as measured by the modified Rankin Scale. Three multivariate models incorporating clinical, demographic, and MRI/EEG variables explained up to 53% of the variance in mRS at discharge (*p* < 0.001), outperforming models based solely on CSF parameters. Key predictors of poor functional outcome included ‘altered consciousness’ (OR 7.08, *p* < 0.001), higher ‘mRS at admission’ (OR 0.03–0.07 across categories, *p* < 0.001), ‘focal/generalized EEG slowing’ (OR 9.97, *p* < 0.001), ‘epileptiform EEG activity’ (OR 17.49, *p* < 0.001), ‘MRI: myelitis’ (OR 16.44, *p* = 0.004), and ‘intrathecal IgM synthesis’ (OR 8.93, *p* = 0.018). Conversely, ‘longer hospitalization’ (OR 0.13–0.17 for different intervals, *p* < 0.006) and ‘intrathecal IgG synthesis’ (OR 0.05, *p* = 0.03) were associated with more favorable outcomes. Despite the single-center and retrospective aspects of this study, our findings underscore a multifactorial pattern of outcome determinants in infectious encephalitis, highlighting the prognostic relevance of initial neurological status, electrophysiological abnormalities, and neuroimaging features.

## 1. Introduction

Encephalitis is a potentially life-threatening inflammatory condition of the brain parenchyma caused by a wide range of infectious and non-infectious etiologies. Common infectious agents include *Herpesviridae* family members, such as *herpes simplex virus type 1* (HSV1) in 13.8% and *varicella-zoster virus* (VZV) in 0.4% of all cases, according to an epidemiological study [1]. Due to growing globalization, the original geographical distribution of certain viruses *(West Nile virus*, *Parechovirus*, *tick-borne meningoencephalitis virus*, and *Japanese encephalitis virus)* is changing and may lead to an increase in encephalitis cases [2]. Despite advances in pathogen detection with proposed high-throughput DNA sequencing (e.g., polymerase chain reaction (PCR) and next-generation sequencing techniques), the underlying etiology remains unidentified in up to 40% to 60% of aseptic encephalitis, implicating a substantial proportion of non-bacterial infectious (i.e., virus, fungus) or autoimmune (paraneoplastic, postinfectious) causes [3,4,5]. This diagnostic uncertainty presents challenges for targeted treatment and prognostic assessment. Furthermore, there is a large field of post-infectious, autoimmune-mediated causes of encephalitis (acute-demyelinating encephalomyelitis (ADEM)), as recently reported after coronavirus 19 disease (COVID-19) and its vaccination [6].

Patients with encephalitis, especially from the *Herpesviridae* family, experience exceedingly high rates of short- and long-term sequelae with a mortality rate of 21.8%, according to an epidemiological study [1]. Despite advances in diagnostic techniques and early therapeutic interventions, especially for HSV1, encephalitis remains associated with substantial long-term cognitive decline in 30% to 70%, with significant impact on functional outcome [7]. While the clinical and functional burden of encephalitis is well recognized, data on outcome predictors at the time of admission, especially in real-world clinical settings, remain limited. Studies on encephalitis patients, including multifaceted diagnostics and especially a full CSF data report with novel biomarkers, e.g., C-X-C-motif chemokine ligand 13 (CXCL13), are scarce. That is why we intended to deeply investigate the role of different clinical, demographic, and diagnostic data on the outcome of encephalitis patients.

In this retrospective single-center study, we aimed to characterize the spectrum of infectious encephalitis cases treated at the University Hospital of Ulm between 2014 and 2024 and to identify various clinical, demographic, neuroimaging, EEG, and CSF parameters associated with short-term functional outcomes, as measured by the modified Rankin Scale (mRS), even on admission and discharge. This study is also the first to integrate a full data CSF report with CSF-CXCL13 in outcome analysis in infectious encephalitis cases.

## 2. Materials and Methods

### 2.1. Patient Selection Process

**Figure 1:**
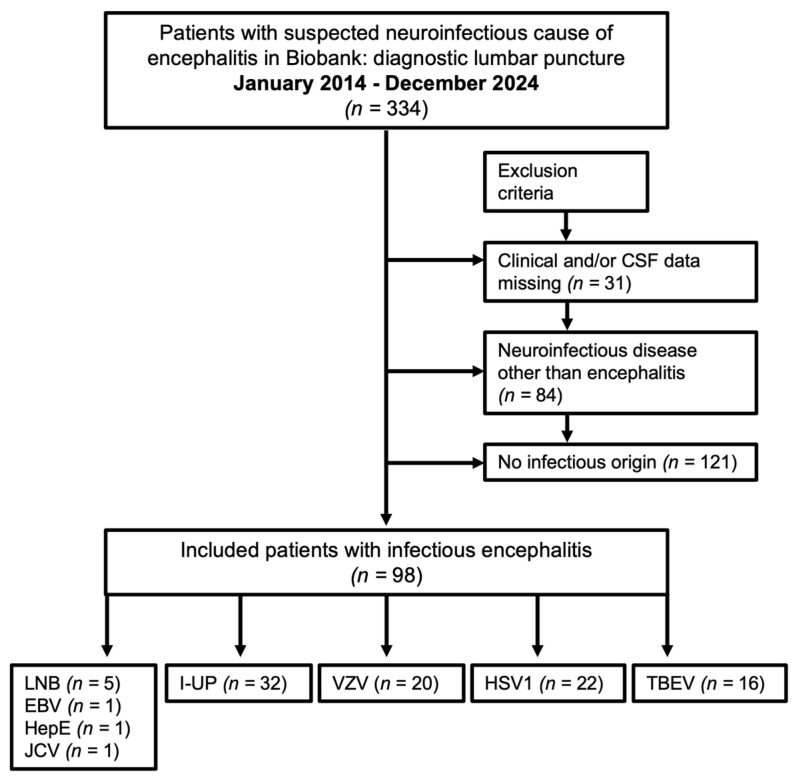
Patient selection process. The patient selection process is described in this flowchart. Due to the suspected neuroinfectious cause of encephalitis, 334 patients in the neurological department of the University Hospital of Ulm received a lumbar puncture. They all consented to the scientific use of their clinical data and biosamples. According to the above exclusion criteria, 98 patients were included in this monocentric retrospective study. CSF: cerebrospinal fluid, TBEV: tick-borne meningoencephalitis virus, HSV1: herpes simplex virus type 1, VZV: varicella-zoster virus, EBV: Epstein–Barr virus, HepE: hepatitis E virus, JCV: John-Cunningham virus, LNB: Lyme neuroborreliosis, I-UP: neuroinfectious diseases of unknown pathogen.

This retrospective and monocentric study included all patients (age ≥ 16 years) in the University Hospital of Ulm (Germany) from January 2014 to December 2024 with encephalitis, meningoencephalitis, and encephalomyelitis according to their medical records and discharge codes. Encephalitis was defined according to the clinical case definition of the International Encephalitis Consortium [8]. All patients had encephalopathy for at least 24 h (main criterion), defined as a change in their mental status/consciousness with no other explanation (e.g., toxins, severe systemic infection/sepsis, or metabolic disorders). In addition, at least two of the following minor criteria were added: fever ≥ 38 °C; MRI findings suggestive of encephalitis (mesial temporal/white matter/brainstem lesions and deep gray matter lesions); white blood cell count in the CSF analysis ≥ 5/µL; new focal neurological findings; EEG (electroencephalogram) findings compatible with encephalitis (focal/generalized slowing, epileptiform abnormalities, and epileptic status); and new onset seizures (partial/generalized or status epilepticus; clinical or EEG confirmation). Meningoencephalitis was assumed for encephalitis patients with intense headaches and signs of meningeal involvement (meningism, contrast enhancement of the meninges in MRI). During the selection process, we included all encephalitis patients with suspicion of infectious cause who received a lumbar puncture for diagnostic purposes and consented to the scientific use of their samples, clinical, and diagnostic data (*n* = 334; Figure 1). We had to exclude 84 patients due to neuroinfectious diseases other than encephalitis (e.g., meningitis, infectious cranial nerve palsies), 121 patients because encephalitis or encephalomyelitis was not of infectious origin, and 31 patients due to the lack of the full CSF report. The whole study cohort included 98 patients with *tick-borne meningoencephalitis* (*n* = 16; TBEV), HSV1 encephalitis (*n* = 22), VZV (meningo-) encephalitis (*n* = 20), *Epstein–Barr virus* encephalitis (*n* = 1; EBV), *hepatitis E virus* encephalitis (*n* = 1), *John-Cunningham virus* encephalitis (*n* = 1; JCV), and definite *Lyme neuroborreliosis (n* = 5; LNB) [9]. Despite direct and indirect pathogen screening and testing for intracellular or surface antibodies (methods described in Section 2.4 and Section 2.5), no pathogen could be detected in 32 patients (I-UP, neuroinfectious diseases of unknown pathogen). However, based on clinical symptoms and laboratory data (fever, leucocytosis in blood and CSF, elevation of C-reactive protein, and disruption of the blood–CSF barrier), a pathogen-induced infection appears to be more likely [10].

### 2.2. Clinical and Demographic Parameters

All patients underwent clinical examination. In addition to the demographic data (age, gender, time from symptom onset to LP/initiation of anti-infective treatment, and duration of hospitalization), the neurological symptoms (focal neurological deficits, e.g., paresis, aphasia, and ataxia) and the modified Rankin Scale (mRS) at admission and discharge were documented in the letter of admission/discharge. The modified Rankin Scale represents a widely used tool to reflect functional outcomes in any disease [7]. The main symptom of encephalitis, encephalopathy, was defined as having altered/loss of consciousness (somnolence, sopor, and coma) or altered mental status (disorientation and confusion). Changes in behavior or personality were included as psychiatric features. We also recorded the type of epileptic seizure (focal, generalized, or status epilepticus) with or without EEG confirmation. Immunosuppression was assumed in the case of HIV/AIDS infection, chemotherapy, or long-term immunosuppressive treatment. Directly after LP, all patients received empirical anti-infective therapy with acyclovir, ceftriaxone, and/or ampicillin/sulbactam until we obtained further results of the direct/indirect pathogen detection. For this reason, we considered symptom onset to LP equivalent to time of therapy initiation.

### 2.3. CSF Analysis

All CSF and serum samples were handled according to the guidelines of the German Societies for Cerebrospinal Fluid Diagnostics and Clinical Neurochemistry and Neurology (DGLN; DGN), respectively [11]. The white blood cells (WBC) in CSF were counted using the Fuchs–Rosenthal chamber (normal value ≤ 4 leucocytes/µL). CSF–lactate was measured photometrically (normal range 1.2–2.4 mmol/L) using AU400/AU680 Clinical Chemistry Analyzers (Olympus/Beckman Coulter, Krefeld, Germany). Albumin (mg/L), total protein (normal range 200–500 g/L), and immunoglobulins M, A, and G (mg/L) in CSF and serum were measured using a nephelometer (BN Prospec nephelometer, Siemens, Munich, Germany). We calculated the CSF/serum albumin quotient (Qalb) to assess the blood–CSF barrier function. The upper limit of the Qalb is age-dependent, so we calculated Qlim(alb) for each patient according to the following formula: Qlim(alb) = (4 + age/15) × 10^−3^ [12]. A blood–CSF barrier dysfunction was assumed for Qalb > Qlim(alb) [13]. Oligoclonal IgG bands in CSF and serum were detected using isoelectric focusing. We built CSF/serum quotients of each immunoglobulin (QIgM, QIgA, and QIgG) and calculated their upper limits (Qlim(Ig)) against Qalb according to Reiber’s revised hyperbolic function [14]. For CSF/serum Ig quotients > Qlim(Ig), we assumed intrathecal synthesis of the respective immunoglobulin and calculated the intrathecal fraction [14].

### 2.4. Direct and Indirect Pathogen Detection

Directly after lumbar puncture, the CSF was analyzed for the following viral and bacterial pathogens using either microscopic/cultural evidence or a nested polymerase chain reaction (PCR) multiplex panel (BioFire^®^ FilmArray^®^, bioMérieux, Marcy-l’Étoile, France): *Herpes simplex virus types 1 and 2*, *human herpes virus type 6*, *human Parechovirus*, *varicella-zoster virus*, *enterovirus*, *cytomegalovirus*, *Escherichia coli K1*, *Haemophilus influenzae*, *Listeria monocytogenes*, *Neisseria meningitidis*, *Streptococcus agalactiae*, *and Streptococcus pneumoniae. Furthermore*, *PCR was performed for tick-borne meningoencephalitis virus*, *Epstein–Barr virus*, *hepatitis E virus*, *and John-Cunningham virus*. We used ELISA (enzyme-linked immunosorbent assay) according to the manufacturer’s instructions (Gold Standard Diagnostics Europe (former Genzyme Virotech), Rüsselsheim, Germany) for the indirect pathogen detection. Antibody levels of IgM and IgG in CSF and serum against HSV1, HSV2, VZV, EBV, CMV, TBEV, and Borrelia burgdorferi (Bb) were determined. We calculated each IgG antibody level’s specific CSF/serum antibody index (AI) to determine its possible intrathecal synthesis. Therefore, we used the following formula: AI = QIg_spec_/QIg_total_ for QIg_total_ < Qlim(Ig) (upper reference limit for QIg as calculated above) and QIg_spec_/Qlim(Ig) for QIg_total_ > Qlim(Ig). We assumed intrathecal synthesis for specific AI values above 1.5.

### 2.5. Intracellular and Surface Autoantibodies

If no pathogen could be detected using the direct and indirect analysis methods described above, we tested for intracellular and surface antibodies in CSF and serum. This test was performed using cell-based assays, line blots, and indirect immunofluorescence. The assays were conducted on unfixed sections of brain, nerve, pancreas, and primate intestines (all Euroimmun, Lübeck, Germany). The tested antibodies were against CV2 (CRMP5), Amphiphysin, Ri, PNMA2 (Ma-2/Ta), Yo, Hu, recoverin, SOX1, Zic4, titin, GAD65, Tr (DNER), glutamate-receptor type (NMDA, AMPA1/2), leucine-rich glioma-inactivated protein 1 (LGI1), contactin-associated protein 2 (CASPR2), DPPX, and GABAB1 receptor. In cases of encephalomyelitis, we also tested for MOG antibodies using live cell-based assays as previously described [15].

### 2.6. CSF-CXCL13

Upon lumbar puncture, samples were centrifuged at 2000× *g* for 10 min and then stored at +2 °C to +8 °C until further analysis. CSF-CXCL13 (C-X-C-motif chemokine ligand 13) was regularly measured after ≤6 days using an ELISA according to the manufacturer’s instructions (until January 2018 Quantikine, R&D Systems, Minneapolis, Minnesota; since February 2018 Euroimmun, Lübeck, Germany). Both ELISAs correlated very well (r = 0.98) according to an in-house method comparison in 40 patients (also specified from Euroimmun). The assay range was 4.6 pg/mL–500 pg/mL for Euroimmun and 7.8 pg/mL–500 pg/mL for Quantikine, R&D Systems. CSF-CXCL13 levels < 10 pg/mL were generally considered normal. CSF-CXCL13 is neither age- nor gender-dependent [10].

### 2.7. MRI Examination

Each patient with encephalopathy received an MRI (magnetic resonance imaging) scan of the brain with or without contrast. In special cases, e.g., paraparesis, we also performed an MRI of the spinal cord. Findings included T2-weighted/FLAIR (fluid-attenuated inversion recovery) abnormalities involving the white matter, cortical gray matter, deep gray matter (e.g., basal ganglia and thalamus), mesial temporal lobes, and/or brainstem. Furthermore, we could detect DWI/ADC (diffusion-weighted imaging/apparent diffusion coefficient) abnormalities regarding ischemic lesions or contrast enhancement of any involved structure.

### 2.8. Statistics

**Figure 2:**
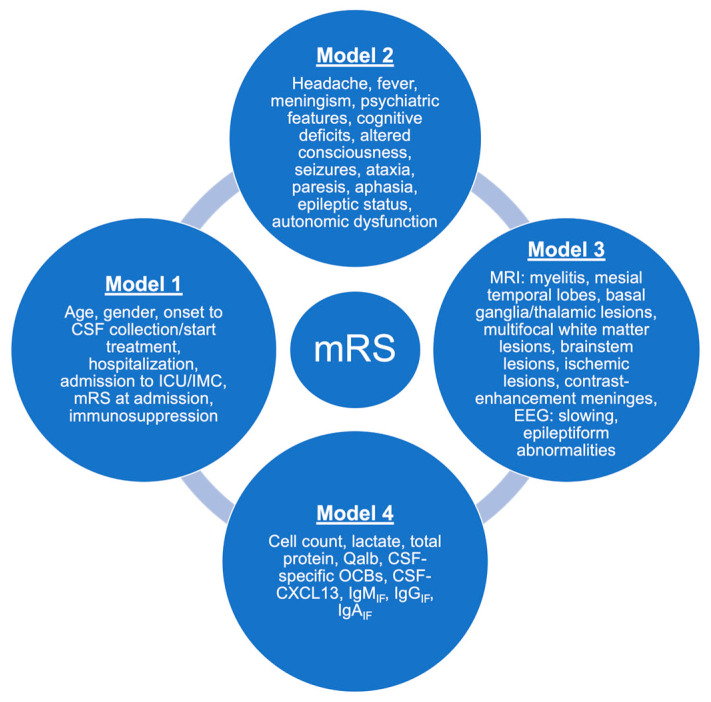
Model 1–4 for ordinal logistic regression analysis. We built four models for the ordinal logistic regression analysis. Model 1 represents demographical data; Model 2 is composed of clinical symptoms; Model 3 consists of instrumental diagnostic data; and Model 4 represents CSF findings. The modified Rankin Scale (mRS) at discharge represents the outcome parameter of this study. ICU: intensive care unit; IMC: intermediate care unit; MRI: magnetic resonance imaging; EEG: electroencephalogram; CSF: cerebrospinal fluid; IgG_IF_, IgM_IF_, and IgA_IF_: intrathecal fraction of IgG, IgM, and IgA; OCBs: CSF-specific oligoclonal IgG bands; Qalb: CSF/serum albumin quotient.

We assumed normal distribution due to the total sample size of *n* > 30 (*n* = 98 encephalitis patients in our study) [16]. Therefore, we used mean value and standard deviation to describe continuous variables (age, time since onset to LP, days of hospitalization, leucocyte count, lactate, total protein, Qalb, and CSF-CXCL13). Discrete variables are given as absolute and relative frequencies (gender, pathogens, mRS at admission and discharge, different clinical symptoms, MRI data, EEG data, CSF-specific oligoclonal IgG bands, intrathecal IgM/IgA/IgG synthesis, and different outcome parameters). We built the following four models: Model 1 ‘Demographic data’, Model 2 ‘Clinical data’, Model 3 ‘MRI and EEG findings’, and Model 4 ‘CSF findings’, rendering all data collected at a patient’s first admission. The multivariate models are illustrated in Figure 2. An ordinal logistic regression analysis was performed using the proportional odds model (cumulative logit model) for the outcome parameter “modified Rankin Scale at discharge” (mRS). This model was chosen because the mRS is an ordinal variable with several ordered categories. The Nagelkerke R^2^, a pseudo R^2^ based on the likelihood calculation, was calculated to assess the quality of the model and enable better comparability between models. Higher values of Nagelkerke R^2^ indicate a better fit of the model to the data. The effect sizes of the independent variables were reported as odds ratios (OR) with associated 95% confidence intervals (CI). An OR > 1 indicates that the predictor is associated with a higher probability of a worse outcome (higher mRS value), while an OR < 1 indicates an association with a more favorable outcome (lower mRS value). The significance of the predictors was tested using the Wald test. In this study, *p*-values < 0.05 were considered as statistically significant. We did not perform any adjustment for age or gender, as we also integrated these two variates into the model to investigate their influence on outcome. The missing data were automatically excluded from the regression analysis by the statistics program IBM^®^ SPSS^®^ Statistics version 28.0.1.0 for macOS (Armonk, NY, USA) using pairwise exclusion. We used Prism version 10.3.0 for macOS (GraphPad Software Inc., La Jolla, CA, USA) for the graphs.

## 3. Results

### 3.1. Patient Characteristics

We included 98 patients with (meningo-) encephalitis with a mean age of 64.13 years (SD = 18.85). The majority was male (*n* = 55; 56.1%). All demographic characteristics are displayed in Table 1. Eight (8.2%) cohort patients were immunosuppressed due to chemotherapeutical treatment or immunosuppressant intake. The mean time from the onset of symptoms to lumbar puncture (LP) was 18.63 days (SD = 75.24). In HSV1 and VZV encephalitis, an LP was performed after 5.23 (mean value ± SD 4.12) days and 6.85 (mean value ± SD 8.89) days, respectively. In contrast, in the five cases of encephalitis due to Lyme neuroborreliosis, the range was 28 to 730 days (mean value 200.2 days ± SD 297.14). Forty-two patients (42.9%) were transferred to the intensive care unit/intermediate care unit (ICU/IMC). The mean time of hospitalization was 23.1 days (SD = 16.23). According to the modified Rankin Scale (mRS), 36.7% (*n* = 36) had an mRS of 4 at LP, 24.5% (*n* = 24) achieved three points, 19.4% had an mRS of 2 (*n* = 19), 17.3% had five points (*n* = 17), and only 2.0% (*n* = 2) had an mRS of 1.

### 3.2. Clinical and Diagnostic Data

Cognitive deficits upon the physician’s examination at presentation were recorded in 77.6% of the cases (*n* = 76) (Table S1). Due to the retrospective nature of this study, a standardized neuropsychological examination was not carried out in the first few days after presentation or before discharge. In addition, fifty-nine patients had headaches (60.2%), forty-nine suffered from altered consciousness (50.0%), forty-eight had fever (49.0%), and forty-four had focal or generalized seizures (44.9%). Regarding the focal neurological deficits, 21.4% of the patients (*n* = 21) had aphasia, and 17.3% (*n* = 17) had paresis. Each patient in our cohort received an MRI of the brain at admission and before the lumbar puncture (Table S2). In 26.5% (*n* = 26) and 21.4% (*n* = 21), we detected lesions in the mesial temporal lobes and generalized white matter with or without contrast enhancement. In addition, 10.2% (*n* = 10) had involvement of the basal ganglia or thalamus. In 12.0% (*n* = 6) of the cases who received a spinal MRI (*n* = 50), we also detected myelitis. Two of those had TBEV with encephalomeningomyelitis, one patient had encephalomyelitis due to an EBV infection, and another patient had encephalomyelitis due to LNB. We also detected two patients with encephalomyelitis of unknown pathogen. We assumed an infectious origin due to the presence of fever, high pleocytosis and blood–CSF barrier disruption in the CSF report, meningeal involvement, and a rapid improvement of the symptoms after starting anti-infective therapy. Seventy-four (75.5%) patients received an EEG in the emergency room (Table S2). Focal or generalized slowing was recorded in 44.6% (*n* = 33/74). An EEG confirmed an epileptic status in 27.0% of the patients (*n* = 20/74), with 35.1% (*n* = 26/74) having epileptiform abnormalities in the EEG. Each patient also received a lumbar puncture at first presentation. The mean leucocyte count was 110.05 cells/µL (SD = 128.49) with a mean lactate of 2.47 mmol/L (SD = 0.90) and a mean total protein of 1140.01 mg/L (SD = 998.03) (Table S3). According to Reiber’s graph, 38.8% (*n* = 38) had CSF-specific oligoclonal IgG bands according to IEF and 22.4% (*n* = 22) an intrathecal IgM synthesis. We also measured CSF-CXCL13 in 54 patients (55.1%). The mean value was 301.7 pg/mL (SD = 1017.93).

### 3.3. Predictors for Outcome

**Figure 3:**
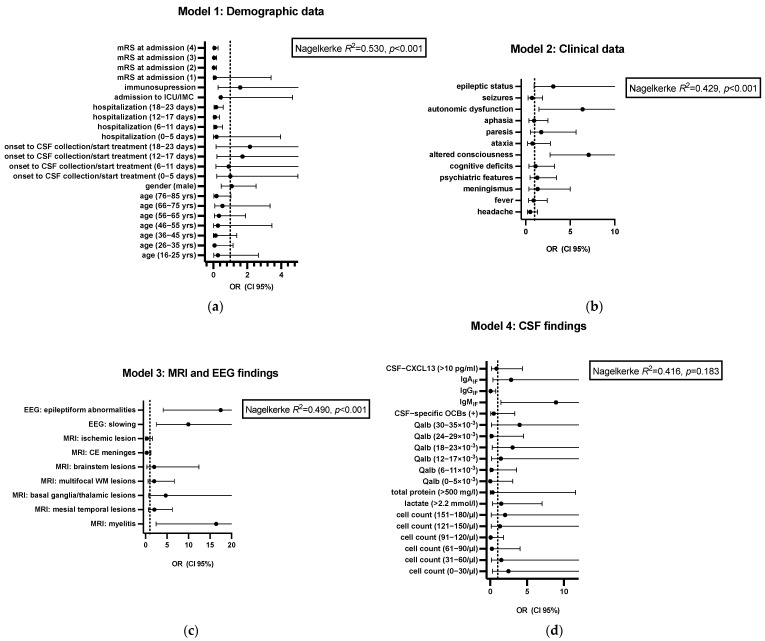
Results of the ordinal logistic regression analysis regarding outcome in encephalitis patients. The results of the ordinal logistic regression analysis of the four models ((**a**) demographic data, (**b**) clinical data, (**c**) MRI and EEG findings, and (**d**) CSF findings) regarding outcome in 98 encephalitis patients are presented in forest plots. The values are given as odds ratio (OR) with its 95% confidential interval (95% CI). Nagelkerke R^2^ represents the extent of the variance with assumed significance for *p*-values < 0.05. mRS: modified Rankin Scale; ICU: intensive care unit; IMC: intermediate care unit; MRI: magnetic resonance imaging; EEG: electroencephalogram; CE: contrast enhancement; WM: white matter; CSF: cerebrospinal fluid; IgG_IF_, IgM_IF_, and IgA_IF_: intrathecal fraction of IgG, IgM, and IgA; OCBs: CSF-specific oligoclonal IgG bands; Qalb: CSF/serum albumin quotient.

Seventy patients (71.4%) had a good outcome according to mRS 0–2 (Table S4). Eight patients died, leading to a mortality rate of 8.2%. The other eighteen (18.3%) patients had poorer mRS 3–5 outcomes. According to the remaining symptoms, 30.6% (*n* = 30) of the surviving patients had persisting cognitive deficits according to the physician’s examination (e.g., examination of orientation to time, place, situation, and person; naming objects; and memorizing and repeating three terms), 17.3% (*n* = 17) had any focal neurological deficit, and even 11.2% (*n* = 11) had persisting altered consciousness/mental status. We performed an ordinal logistic regression analysis to determine factors associated with the functional outcome (mRS at discharge). In addition, we calculated the extent of the variance explained by the four models ‘Demographic data’, ‘Clinical data’, ‘MRI and EEG findings’, and ‘CSF findings’ (Figure 3). The results are displayed in Figure 3 and Table 2.

The first model, ‘Demographic data’, indicated that approximately 53% of the variance in the modified Rankin Scale (mRS) at discharge was highly significantly explained by the included predictors (*p* < 0.001). Furthermore, the clinical symptoms at presentation could explain 42.9% of the variance (*p* < 0.001). The third model, ‘MRI and EEG findings’, significantly explained 49% of the variance (*p* < 0.001), outperforming the CSF data model (*p* = 0.183). In the next step, we wanted to identify single variates of each model associated with functional outcome at discharge, measured by the mRS. The reference category for the outcome variable was mRS = 6 (death), i.e., all other estimates were calculated based on mRS = 6 (Table 2). For categorical independent variables (e.g., binary variables such as ‘headache: yes’ vs. ‘headache: no’), we omitted one category from the analysis to serve as the reference factor (in our case, ‘headache: no’). Similarly, we omitted the highest category for ordinal or grouped continuous variables (such as ‘hospitalization’ in days) because this served as the reference group. The analysis revealed that length of hospitalization (6–23 days) had a significant association with mRS at discharge (Figure 3). Patients in intermediate hospitalization categories (2: 6–11 days; 3: 12–17 days; 4: 17–23 days) had a significantly higher likelihood of a favorable outcome (i.e., lower mRS at discharge) compared to those in the reference group (*p* < 0.01). Another strong predictor was the mRS score at admission. Higher baseline disability scores (mRS = 2–4) were associated with a significantly lower probability of a favorable outcome (all *p*-values < 0.001). In contrast, age, gender, immunosuppression status, ICU/IMC admission, and the time from symptom onset to CSF sampling did not show statistically significant associations with the mRS at discharge in this model. Although not statistically significant (16–25 yrs: *p* = 0.064; 36–45 yrs: *p* = 0.092; 76–85 yrs: *p* = 0.055), younger age at admission seems to be protective. Regarding the clinical symptoms, patients with impaired consciousness on admission were significantly more likely to have a poorer outcome (higher mRS) on discharge than patients without (*p* < 0.001, OR 7.08 (95% CI 2.73–18.32)). Patients with autonomic dysfunctions (e.g., circulatory and temperature regulation dysfunction, bladder and bowel dysfunction) had a significantly poorer mRS at discharge (*p* < 0.001, OR 6.39 (95% CI 1.50–27.30)). Epileptic status at admission tended to result in worse outcomes at discharge without reaching significance (*p* = 0.065, OR 3.10 (95% CI 0.93–10.34)). All other neurological symptoms at admission had no significant effect on mRS at discharge (*p* > 0.05). Patients with EEG slowing (either focal or generalized) had a significantly higher probability of a poorer outcome regarding the mRS (*p* < 0.001, OR 9.97 (95% CI 2.54–39.10)). Epileptiform abnormalities in the EEG were even associated with a worse outcome according to the mRS (*p* < 0.001, OR 17.49 (95% CI 4.12–74.44)). Considering the MRI findings, myelitis resulted in a poorer outcome (*p* = 0.004, OR 16.44 (95% CI 2.43–111.39)). There was a tendency towards a poorer outcome for patients with deep gray matter lesions (basal ganglia, thalamus) without reaching statistical significance (*p* = 0.097). Regarding the CSF findings, the only significant predictor for worse outcomes in our cohort was the presence of an intrathecal IgM synthesis according to the Reiber’s formula (*p* = 0.018, OR 8.93 (95% CI 1.44–55.15)). In contrast, according to Reiber’s formula, an intrathecal IgG synthesis predicted a better outcome (*p* = 0.03, OR 0.05 (95% CI 0.003–0.75)). No other CSF findings had a significant impact on the outcome (*p* > 0.05). A table with all significant and non-significant outcome factors can be additionally found in the Supplementary Materials section (Table S5).

## 4. Discussion

This is one of the first studies to investigate the prognostic role of broad demographic data, neurological symptoms, MRI/EEG data, and complete CSF data reports (incl. CSF-CXCL13) in a large and heterogenous cohort of encephalitis patients. Our results show that altered consciousness and autonomic dysfunction, focal/generalized slowing or epileptiform abnormalities in the first EEG, specific MRI (myelitis), higher scores on the modified Rankin Scale at admission, and intrathecal IgM synthesis in the CSF analysis according to Reiber’s graph were associated with poorer outcomes. Longer days of hospitalization and an intrathecal IgG synthesis were associated with lower mRS. The explained variance differed across models, with clinical characteristics accounting for a smaller proportion of variance than demographic data and MRI/EEG findings, contributing more substantially to the overall model fit. The overall outcome was quite good, with 71.4% of the patients having an mRS of 0–2 at discharge, similar to a recent Chinese study [17]. The average mortality rate in our cohort was 8.2%. This aligns with the global encephalitis mortality rate of 5–15% in other studies [17,18]. We could detect a pathogen in 67.3% of all encephalitis cases, similar to studies published previously [4,19,20]. The most commonly detected sporadic cause of infectious encephalitis is herpes simplex virus type 1 [18]. In our study, HSV1 accounted for 22.4% of all cases, followed directly by VZV (20.4%). In contrast to other studies, our study did not focus on special entities, e.g., *Herpesviridae* (mainly HSE), and neither were we restricted to patients admitted to ICU or IMC [21,22,23,24,25,26]. Older age, immunocompromised state, and ICU admission accounted for adverse outcomes in previous studies [20,21,26]. In this study, 42.9% of the patients in our cohort were admitted to ICU/IMC, similar to other studies [20]. In our study, a higher mRS and older age at admission were associated with poorer outcomes, reflecting the expected clinical course. Interestingly, longer duration of hospitalization was associated with better outcome in our cohort. The inpatient stay in our cohort was longer (mean value 23.10 days (± SD 16.23)) in contrast to other studies, which varied from mean values of 11.2 to 15 days [1,20]. Previous studies have reported no statistically significant correlation between outcome and hospitalization days [27].

Neurological symptoms in general accounted for an extent of 42.9% of the variance. Altered consciousness and autonomic dysfunction were identified as significant predictors for poorer outcomes. Most prospective studies used the Glasgow Coma Scale (GCS) score for addressing consciousness, and some found associations with poorer outcomes and lower GCS scores [20,21,22,26]. Due to the retrospective nature of our study, we did not obtain the data on the GCS and, therefore, used a binary variable for ‘altered consciousness’ (i.e., ‘yes’ or ‘no’). None of the previously published studies have addressed the role of autonomic dysfunction on outcomes in acute infectious encephalitis so far. However, it has already been shown that patients with acute autoimmune encephalitis (mainly anti-N-methyl-D-aspartate receptor- and anti-contactin-associated protein-2 receptor-associated) may develop different types of dysautonomia (e.g., cardiac, vasomotor, urogenital) [28,29].

In our study, 65.2% of the patients had abnormal MRI findings with cerebral FLAIR-/T2-weighted lesions with or without T1-weighted contrast enhancement in the white matter or deep gray matter involving mesial temporal lobes, basal ganglia, thalamus, or the brainstem. Interestingly, only specific MRI findings (myelitis) were significantly associated with poorer outcomes. A tendency towards significant association with poorer outcomes was detected for deep gray matter lesions in the basal ganglia and thalamus. However, the model ‘MRI and EEG findings’ explained 49% of the variance. This is in contrast to former studies, particularly on HSV1 encephalitis (HSE) in ICU, which showed that either focal cortical parenchymal hyperintensities, lesions involving >3 lobes, DWI abnormalities in the left thalamus, or generalized cerebral edema were significantly associated with poorer outcomes [26,30,31,32]. Explanations for the different findings might be that we included different viral/bacterial etiologies for encephalitis and the calculations. Furthermore, we did not only include ICU patients in our analysis.

In addition to the MRI findings, we found that any changes in the EEG at admission (focal or generalized slowing, epileptiform abnormalities) were associated with poorer mRS in our cohort. Focal slow waves and continuous delta waves in EEG were associated with adverse outcomes in a former study [32]. Status epilepticus only reached a tendency towards significance concerning worse outcomes (*p* = 0.065). Although 44.9% of all patients had seizures at admission, this was not associated with outcome. This aligns with the study of Singh et al., which also showed a lack of significance regarding epileptic status and seizures but contradicts previously published studies [20,22,26,33]. Epileptic seizures are common in infectious encephalitis, especially HSE [23]. Up to 75% of the HSE patients have epileptiform abnormalities in EEG in the first 24 h [23]. However, epileptic seizures were not associated with a higher three-month mortality rate following the acute stage of encephalitis [33].

Interestingly, CSF data at admission did not significantly explain the extent of the variance. CSF collection is pivotal in patients with suspicion of encephalitis for differential diagnostic and treatment purposes [5,18,34]. One study showed that the combination of fever, nausea, erythrocyte sedimentation rate <17 mm, and moderate pleocytosis in CSF (WBCC 5–100 × 10^6^/L) had the highest diagnostic accuracy to discriminate encephalitis in a group of encephalopathy patients [34]. We found a mean white blood cell count in our cohort of 110.05 leucocytes/µL (±SD 128.49). Former studies found no significant difference regarding leukocyte count and CSF-specific oligoclonal bands in infectious encephalitis [35,36]. However, there is a tendency towards higher total protein levels in HSE and VZV CNS infections [37,38]. Studies on CSF data addressing the outcome of infectious encephalitis patients of any cause are scarce. Thus far, the primary focus has been on raised intracranial and CSF pressure (>400 mmH_2_O) with poor outcome and a higher risk for mechanical ventilation in the ICU [17,22]. One study also found that CSF polymorphonuclear cell count is associated with adverse outcomes in patients with acute viral encephalitis [20]. However, we, also in line with other studies, found that neither white blood cell count nor total protein in CSF were significant predictors of outcomes in our cohort of encephalitis patients [17,39].

CXCL13 serves as a chemoattractant for B-lymphocytes and B-helper follicular T-lymphocytes to direct them into the germinal centers of B-cell follicles of secondary or tertiary lymphoid organs [40]. CXCL13 is mainly produced by mononuclear antigen-presenting cells upon interactions of Toll-like receptor 2 on monocytes with special outer surface lipoproteins on spirochetes [41]. CSF-CXCL13 levels were elevated in our cohort with a mean value of 301.70 pg/mL (± SD 1017.93), similar to cases of meningoencephalitis patients in Fujimori et al. [42]. According to former studies, CSF-CXCL13 is not a reliable marker for discriminating various viral CNS infections, but it is a marker of acute disease, especially very early Lyme neuroborreliosis (LNB) [10,42,43,44]. Nevertheless, CSF-CXCL13 is significantly elevated in LNB and serves mainly as a diagnostic and treatment response marker in LNB, which showed a sharp decline after starting the antibiotics [10,45,46]. CSF-CXCL13 was significantly higher in patients with encephalitis compared to meningitis [10,42,43]. In contrast to our study, higher CSF-CXCL13 values at the first LP and follow-up indicated a more complicated disease course and served as a prognostic marker [42,43]. However, these studies had only small cohorts of meningoencephalitis patients (*n* = 8 and *n* = 5, respectively) in contrast to ours [42,43]. For the clinical value, single CSF-CXCL13 values should be treated with caution. However, if there is a decrease in follow-up measurements, it can also be a therapy response marker in diseases other than Lyme neuroborreliosis.

Our study showed prognostic evidence for the quantitative fraction of intrathecal IgM (IgM_IF_) and IgG (IgG_IF_) according to Reiber’s formulae. IgM_IF_ was associated with poorer outcomes in our encephalitis cohort than IgG_IF_. In a recently published study, an ongoing TBEV-specific IgM production with higher proportions of intrathecal IgM synthesis than IgG production was associated with adverse outcomes [47]. Consequently, lower intrathecal IgG fractions at admission correlated with worse sequelae due to an assumed delayed seroconversion and intrathecal conversion from IgM to IgG production [48]. Another possible explanation might be the link of intrathecal IgM as the isotype of the immunoglobulins, which is mainly associated with complement activation, especially C3. Increased values of complement proteins in CSF at first presentation were associated with worse outcomes in studies with viral CNS infections, e.g., HSE [49,50].

### Strengths and Limitations

The main strength of our study is that it resembles a broad ‘real-world cohort’ with distinct pathogens. On the other hand, this could also be a limitation due to the masking of pathogen-specific findings. Including various and detailed clinical and demographic data, e.g., MRI, EEG, and CSF findings from admission, was advantageous for understanding the general role of different variates on functional outcome. Furthermore, we were the first to include CSF-CXCL13 as a potential variate affecting the outcome of encephalitis patients. This study also has some limitations inherent to the retrospective design, e.g., the lack of EEG findings and CSF-CXCL13 in 24.5% (*n* = 24) and 44.9% (*n* = 44) of the total cohort (*n* = 98). Concerning the clinical symptoms at admission, not each patient received an EEG in the emergency room. Furthermore, the decision to perform a spinal MRI was made solely based on the patient’s clinical symptoms (e.g., paraparesis). Regarding the statistical analysis, SPSS automatically implemented the pairwise exclusion, retaining the maximum number of valid observations for each analysis and preserving statistical power while minimizing bias introduced by non-random missingness. We decided not to use imputation techniques since the missing variables were not uniformly required for clinical evaluation, and their absence may not be independent of the outcomes or other covariates, violating the assumptions required for valid imputation. Using imputation in such a context could introduce bias. In addition, due to the retrospective design, there was no standardized diagnostic assessment and follow-up period. Likewise, no standardized neuropsychological examination to assess cognitive outcome was carried out during admission, the inpatient stay, or at discharge. This is one of the biggest limitations of this study, as long-term cognitive decline represents one of the most negative outcomes in infectious encephalitis [7]. Several outcome predictors showed wide 95% confidence intervals, indicating a degree of uncertainty in the estimated effects. This likely reflects limited statistical power, variability within the patient population, or sparse data in specific subgroups, all of which reduce the precision and reliability of the inferred associations and highlight the need for larger, multicenter validation studies to improve precision. Additionally, due to the small group size of the sub-diagnoses, no comparison was possible between these.

## 5. Conclusions

In summary, altered mental status, autonomic dysfunction, specific EEG and MRI findings, and CSF-based immunological markers are valuable prognostic indicators in infectious encephalitis. These parameters may support early risk stratification and guide tailored therapeutic approaches. Our results underscore the need for integrated clinical and paraclinical assessment in the acute management of encephalitis.

Future prospective studies addressing probable predictors at admission are warranted in a broader cohort with different viral and bacterial etiologies compared to autoimmune encephalitis. These (multi-center) studies should include standardized diagnostics at admission and follow-up periods; neuropsychological examinations at admission with follow-up assessments; and well-established biomarkers, such as neurofilament light chain and glial fibrillary acidic protein in blood or CSF, to enhance clinical applicability.

## Figures and Tables

**Table 1:** Demographics and clinical data.

Variables	Patients with Encephalitis (*n* = 98)
Age [years], *M* (±SD)	64.13 (±18.85)
Gender (male/female)	56.1%/43.9% (55/43)
Herpesviridae ● HSV1 (*n*) ● VZV (*n*) TBEV (*n*) HepE (*n*) JCV (*n*) EBV (*n*) LNB (*n*) I-UP (*n*)	22.4% (22) 20.4% (20) 16.3% (16) 1.0% (1) 1.0% (1) 1.0% (1) 5.1% (5) 32.7% (32)
Onset to CSF collection/start treatment [days], *M* (±SD)	18.63 (±75.24)
Hospitalization [days], *M* (±SD)	23.10 (±16.23)
Immunosuppression (*n*)	8.2% (8)
Admission to ICU/IMC *(n)*	42.9% (42)
mRS at admission ● 1(*n*) ● 2(*n*) ● 3(*n*) ● 4(*n*) ● 5(*n*)	2.0% (2) 19.4% (19) 24.5% (24) 36.7% (36) 17.3% (17)

SD: standard deviation; M: mean value; HSV1: herpes simplex virus type 1; VZV: varicella-zoster virus; TBEV: tick-borne meningoencephalitis virus; HepE: hepatitis E; JCV: John-Cunningham virus; EBV: Epstein–Barr virus; LNB: Lyme neuroborreliosis; I-UP: infection with an unknown pathogen; CSF: cerebrospinal fluid; ICU/IMC: intensive care unit/intermediate care unit; mRS: modified Rankin Scale.

**Table 2:** Predictors for outcome in encephalitis.

Variables	OR (95% CI)	*p*-Value
Age (26–35 yrs)	0.07 (0.005–1.17)	0.064
Age (36–45 yrs)	0.14 (0.01–1.38)	0.092
Age (76–85 yrs)	0.19 (0.03–1.03)	0.055
Hospitalization (6–11 days)	0.13 (0.03–0.55)	**0.006**
Hospitalization (12–17 days)	0.10 (0.03–0.36)	**<0.001**
Hospitalization (18–23 days)	0.17 (0.05–0.58)	**0.005**
mRS at admission (2)	0.03 (0.005–0.19)	**<0.001**
mRS at admission (3)	0.04 (0.01–0.19)	**<0.001**
mRS at admission (4)	0.07 (0.02–0.28)	**<0.001**
Altered consciousness	7.08 (2.73–18.32)	**<0.001**
Autonomic dysfunction	6.39 (1.50–27.30)	**0.012**
MRI: myelitis	16.44 (2.43–111.39)	**0.004**
MRI: basal ganglia/thalamic lesions	4.70 (0.76–29.22)	0.097
EEG: slowing	9.97 (2.54–39.10)	**<0.001**
Epileptic status	3.10 (0.93–10.34)	0.065
EEG: epileptiform abnormalities	17.49 (4.12–74.44)	**<0.001**
IgM_IF_	8.93 (1.44–55.15)	**0.018**
IgG_IF_	0.05 (0.003–0.75)	**0.030**

Significant results are highlighted in bold. mRS: modified Rankin Scale; MRI: magnetic resonance imaging; EEG: electroencephalogram; IgM_IF_: intrathecal IgM fraction; IgG_IF_: intrathecal IgG fraction; OR: odds ratio; 95% CI: 95% confidential interval.

## Data Availability

The raw data supporting the conclusions of this article will be made available by the authors on request.

## Supplementary Materials

The following supporting information can be downloaded at: https://www.mdpi.com/article/10.3390/life15081313/s1. Table S1: Clinical data at admission; Table S2: Diagnostic findings at admission; Table S3: CSF findings at admission; Table S4: Outcome parameters at discharge; Table S5: All outcome factors, divided into the four models.
