# Supplementary material for: Encephalitis: Predictive Role of Clinical and Diagnostic Data on Outcome—A Monocentric Study
Source: Life (Basel). 2025 Aug 19;15(8):1313. doi: 10.3390/life15081313 (PMC12387950; doi:10.3390/life15081313)
Supplement: Supplementary file 1 [file life-15-01313-s001.zip › life-3767806-supplementary.pdf]

Supplementary Material

Table S1: Clinical data at admission.

	**Patients with encephalitis (n=98)**
Headache (n)	60.2% (59)
Fever (n)	49.0% (48)
Meningism (n)	13.3% (13)
Altered consciousness (n)	50.0% (49)
Psychiatric features (n)	19.4% (19)
Cognitive deficits (n)	77.6% (76)
Seizures (n)	44.9% (44)
Epileptic status (n)	20.4% (20)
Paresis (n)	17.3% (17)
Aphasia (n)	21.4% (21)
Ataxia (n)	11.2% (11)
Autonomic dysfunction (n)	14.3% (14)

Table S2: Diagnostic findings at admission.

	**Patients with encephalitis**
MRI	
– Mesial temporal lobes (n)	26.5% (26/98)
– Basal ganglia/thalamic involvement (n)	10.2% (10/98)
– Brainstem lesions (n)	7.1% (7/98)
– White matter lesions (n)	21.4% (21/98)
– Ischemic lesions (n)	12.2% (12/98)
– Myelitis (n)	12.0% (6/50)
– Meningeal contrast-enhancement (n)	8.2% (8/98)
EEG	
– Focal/generalized slowing (n)	44,6% (33/74)
– Epileptiform abnormalities (n)	35,1% (26/74)
– Normal (n)	20.3% (15/74)
– Not done (n)	24.5% (24/98)

MRI: magnetic resonance imaging, EEG: electroencephalogram.

Table S3: CSF findings at admission.

	**Patients with encephalitis**
Leucocyte count [/µl], *M* (±SD)	110.05 (±128.49); n=98/98
Lactate [mmol/l], *M* (±SD)	2.47 (±0.90); n=98/98
Total protein [mg/l], *M* (±SD)	1140.01 (998.03); n=98/98
Qalb (CSF/S; $x10^{-03}$), *M* (±SD)	17.79 (±18.33); n=98/98
CSF-specific oligoclonal IgG bands (n)	38.8% (38); n=98/98
IgM_{IF} (n)	22.4% (22); n=98/98
IgG_{IF} (n)	13.3% (13); n=98/98
IgA_{IF} (n)	8.2% (8); n=98/98
CSF-CXCL13 [pg/ml], *M* (±SD)	301.70 (±1017.93); n=54/98

CSF: cerebrospinal fluid, M: mean value, SD: standard deviation, Qalb CSF/S: CSF/serum albumin quotient, IgM_{IF}: intrathecal IgM fraction, IgG_{IF}: intrathecal IgG fraction, CXCL13: C-X-C-motif chemokine ligand 13.

Table S4: Outcome parameters at discharge.

	**Patients with encephalitis (n=98)**
mRS at discharge	
– 0 (n)	31.6% (31)
– 1 (n)	23.5% (23)
– 2 (n)	16.3% (16)
– 3 (n)	4.1% (4)
– 4 (n)	7.1% (7)
– 5 (n)	7.1% (7)
– 6 (n)	8.2% (8)
Cognitive deficits (n)	30.6% (30)
Any focal neurological deficit (n)	17.3% (17)
Altered consciousness (n)	11.2% (11)

mRS: modified Rankin Scale.

Table S5: All outcome factors, divided into the four models.

	OR (95% CI)	p-value
**Demographic data**		
Age (16-25 yrs)	.27 (.03-2.66)	.263
Age (26-35 yrs)	.07 (.005-1.17)	.064
Age (36-45 yrs)	.14 (.01-1.38)	.092
Age (46-55 yrs)	.27 (.02-3.46)	.314
Age (56-65 yrs)	.34 (.06-1.91)	.218
Age (66-75 yrs)	.54 (.09-3.34)	.507
Age (76-85 yrs)	.19 (.03-1.03)	.055
Gender (male)	1.08 (.47-2.52)	.851
Onset to CSF collection/start treatment (0-5 days)	1.01 (.20-4.99)	.994
Onset to CSF collection/start treatment (6-11 days)	.91 (.16-5.25)	.919
Onset to CSF collection/start treatment (12-17 days)	1.72 (.21-14.20)	.614
Onset to CSF collection/start treatment (18-23 days)	2.16 (.16-28.90)	.560
Admission to ICU/IMC	1.50 (.48-4.68)	.486
Hospitalization (0-5 days)	.19 (.009-3.97)	.285
Hospitalization (6-11 days)	**.13 (.03-.55)**	**.006**
Hospitalization (12-17 days)	**.10 (.03-.36)**	**<.001**
Hospitalization (18-23 days)	**.17 (.05-.58)**	**.005**
Immunosuppression	1.59 (.28-9.01)	.603
mRS at admission (1)	.09 (.003-3.41)	.198
mRS at admission (2)	**.03 (.005-.19)**	**<.001**
mRS at admission (3)	**.04 (.01-.19)**	**<.001**
mRS at admission (4)	**.07 (.02-.28)**	**<.001**

**Clinical data**		
Headache	.51 (.19-1.43)	.174
Fever	.90 (.33-2.42)	.835
Meningismus	1.35 (.36-5.00)	.655
Psychiatric features	1.30 (.49-3.46)	.599
Cognitive deficits	1.10 (.38-3.22)	.859
Altered consciousness	**7.08 (2.73-18.32)**	**<.001**
Ataxia	.76 (.21-2.78)	.684
Paresis	1.75 (.54-5.68)	.353
Aphasia	.95 (.36-2.52)	.917
Autonomic dysfunction	**6.39 (1.50-27.30)**	**.012**
Seizures	.71 (.26-1.90)	.493
Epileptic status	3.1 (.93-10.34)	.065
**MRI and EEG findings**		
MRI: myelitis	**16.44 (2.43-111.39)**	**.004**
MRI: mesial temporal lobes	2.04 (.67-6.23)	.209
MRI: basal ganglia/thalamic lesions	4.70 (.76-29.22)	.097
MRI: multifocal white matter lesions	2.01 (.60-6.71)	.256
MRI: brainstem lesions	2.00 (.32-12.44)	.456
MRI: CE meninges	.27 (.06-1.26)	.095
MRI: ischemic lesions	.31 (.06-1.60)	.162
EEG: slowing	**9.97 (2.54-39.10)**	**<.001**
EEG: epileptiform abnormalities	**17.49 (4.12-74.44)**	**<.001**
**CSF findings**		
Cell count (0-30/µl)	2.47 (.33-18.82)	.381
Cell count (31-60/µl)	1.51 (.17-13.28)	.710
Cell count (61-90/µl)	.23 (.01-4.06)	.315
Cell count (91-120/µl)	.06 (.002-1.79)	.103
Cell count (121-150/µl)	1.33 (.13-13.11)	.809
Cell count (151-180/µl)	2.03 (.16-26.50)	.589
Lactate (>2.2 mmol/l)	1.49 (.32-7.05)	.611
Total protein (>500 mg/l)	.32 (.009-11.60)	.538
QAlb ($0\text{-}5x10^{-03}$)	.01 (.00008-3.07)	.123
QAlb ($6\text{-}11x10^{-03}$)	.19 (.01-3.58)	.267
QAlb ($12\text{-}17x10^{-03}$)	1.44 (.16-13.21)	.744
QAlb ($18\text{-}23x10^{-03}$)	3.03 (.28-32.75)	.361
QAlb ($24\text{-}29x10^{-03}$)	.20 (.009-4.54)	.314
QAlb ($30\text{-}35x10^{-03}$)	3.99 (.16-98.70)	.397
CSF-specific oligoclonal IgG bands	.47 (.07-3.34)	.450
IgM_{IF}	**8.93 (1.44-55.15)**	**.018**
**IgG_{IF}**	**.05 (.003-0.75)**	**.030**
IgA_{IF}	2.83 (.37-21.76)	.318
CSF-CXCL13 (>10 pg/ml)	.86 (.17-4.36)	.854

Significant results are highlighted in bold. *mRS: modified Rankin Scale, MRI: magnetic resonance imaging, CE: contrast enhancement, EEG: electroencephalogram, IgM_{IF}: intrathecal IgM fraction , IgG_{IF}: intrathecal IgG fraction , OR: Odds ratio, 95% CI: 95% confidential interval, ICU/IMC: intensive care unit/intermediate care unit, CSF:*

cerebrospinal fluid, Qalb CSF/S: CSF/serum albumin quotient, IgM_{IF}: intrathecal IgM fraction, IgG_{IF}: intrathecal IgG fraction, CXCL13: C-X-C-motif chemokine ligand 13.
